# Human umbilical cord-derived mesenchymal stromal cells improve myocardial fibrosis and restore miRNA-133a expression in diabetic cardiomyopathy

**DOI:** 10.1186/s13287-024-03715-2

**Published:** 2024-04-24

**Authors:** Boxin Liu, Yan Wei, Jingjing He, Baofeng Feng, Yimeng Chen, Ruiyun Guo, Matthew D. Griffin, Seán O. Hynes, Sanbing Shen, Yan Liu, Huixian Cui, Jun Ma, Timothy O’Brien

**Affiliations:** 1 Stem Cell Research Center, Hebei Medical University-University of Galway, Hebei Medical University, Hebei Province, 050017 China; 2 Hebei Research Center for Stem Cell Medical Translational Engineering, Hebei Province, 050017 China; 3 Human Anatomy Department, Hebei Medical University, Hebei Province, 050017 China; 4 Regenerative Medicine Institute (REMEDI) at CÚRAM SFI Research Centre for Medical Devices, School of Medicine, University of Galway, Galway, Ireland; 5 Discipline of Pathology, School of Medicine, University of Galway, Galway, Ireland; 6 Department of Endocrinology, Hebei Medical University Third Affiliated Hospital, Shijiazhuang, Hebei 050051 China; 7 Hebei Technology Innovation Center for Stem Cell and Regenerative Medicine, Hebei Province, China; 8 Hebei International Joint Research Center for Stem Cell and Regenerative Medicine, Hebei Province, China

**Keywords:** Diabetes mellitus, Diabetic cardiomyopathy, Mesenchymal stromal cells, Cardiac fibrosis, Micro-RNAs, miRNA-133a, Inflammation

## Abstract

**Background:**

Diabetic cardiomyopathy (DCM) is a serious health-threatening complication of diabetes mellitus characterized by myocardial fibrosis and abnormal cardiac function. Human umbilical cord mesenchymal stromal cells (hUC-MSCs) are a potential therapeutic tool for DCM and myocardial fibrosis via mechanisms such as the regulation of microRNA (miRNA) expression and inflammation. It remains unclear, however, whether hUC-MSC therapy has beneficial effects on cardiac function following different durations of diabetes and which mechanistic aspects of DCM are modulated by hUC-MSC administration at different stages of its development. This study aimed to investigate the therapeutic effects of intravenous administration of hUC-MSCs on DCM following different durations of hyperglycemia in an experimental male model of diabetes and to determine the effects on expression of candidate miRNAs, target mRNA and inflammatory mediators.

**Methods:**

A male mouse model of diabetes was induced by multiple low-dose streptozotocin injections. The effects on severity of DCM of intravenous injections of hUC-MSCs and saline two weeks previously were compared at 10 and 18 weeks after diabetes induction. At both time-points, biochemical assays, echocardiography, histopathology, polymerase chain reaction (PCR), immunohistochemistry and enzyme-linked immunosorbent assays (ELISA) were used to analyze blood glucose, body weight, cardiac structure and function, degree of myocardial fibrosis and expression of fibrosis-related mRNA, miRNA and inflammatory mediators.

**Results:**

Saline-treated diabetic male mice had impaired cardiac function and increased cardiac fibrosis after 10 and 18 weeks of diabetes. At both time-points, cardiac dysfunction and fibrosis were improved in hUC-MSC-treated mice. Pro-fibrotic indicators (α-SMA, collagen I, collagen III, Smad3, Smad4) were reduced and anti-fibrotic mediators (FGF-1, miRNA-133a) were increased in hearts of diabetic animals receiving hUC-MSCs compared to saline. Increased blood levels of pro-inflammatory cytokines (IL-6, TNF, IL-1β) and increased cardiac expression of IL-6 were also observed in saline-treated mice and were reduced by hUC-MSCs at both time-points, but to a lesser degree at 18 weeks.

**Conclusion:**

Intravenous injection of hUC-MSCs ameliorated key functional and structural features of DCM in male mice with diabetes of shorter and longer duration. Mechanistically, these effects were associated with restoration of intra-myocardial expression of miRNA-133a and its target mRNA COL1AI as well as suppression of systemic and localized inflammatory mediators.

**Supplementary Information:**

The online version contains supplementary material available at 10.1186/s13287-024-03715-2.

## Background

Diabetes mellitus (DM) is a metabolic disease resulting in disordered glucose metabolism due to absolute or relative insufficiency of insulin secretion and/or insulin resistance [1–3]. The prevalence of DM is increasing rapidly worldwide. Among its many adverse effects, DM has been recognized as an independent risk factor for heart failure (HF) [4]. Diabetic cardiomyopathy (DCM), one of the target-organ complications of DM, is characterized by abnormal cardiac systolic and diastolic function, myocardial fibrosis and hypertrophy [5–8]. Myocardial fibrosis, an important pathological feature of DCM, is associated with glycosylated collagen deposition as a result of a prolonged hyperglycemic state [9]. MicroRNAs (miRNAs) are small non-coding RNAs with a length of 18–22 nucleotides, which can regulate protein expression at the mRNA level [10]. Alleviation of DCM and myocardial fibrosis by hUC-MSCs may be achieved by regulating the expression of miRNAs [11]. In addition, myocardial fibrosis is usually accompanied by changes in the intra-cardiac expression of a range of fibrosis-related gene products, such as alpha smooth muscle actin (α-SMA), transforming growth factor beta (TGF-β), collagen I, collagen III, suppressor of mothers against decapentaplegic 2 (SMAD2), SMAD3, SMAD4 and fibroblast growth factor 1 (FGF1) [12].

Mesenchymal stromal cells (MSCs) originate from mesoderm and ectoderm in the early development process, and have multi-lineage differentiation potential [13]. Human umbilical cord MSCs (hUC-MSCs) are multifunctional cells that exist in neonatal umbilical cord tissue and have potential for broad clinical applications [14]. It is reported that hUC-MSCs have important anti-apoptotic, anti-fibrotic and anti-inflammatory properties in heart diseases, including DCM [15]. However, most previous studies have almost exclusively focused on the therapeutic effect of certain cells (BM-MSCs) other than UC-derived MSCs and at one time point in the course of DCM [16–18]. These aspects are rarely analyzed in the literature, for example, the optimal timing of therapy and the primary therapeutic effects responsible for the prevention of myocardial fibrosis by systemic administration of hUC-MSCs in the setting of diabetes. Our research addresses several further questions on the therapeutic effect of UC-MSCs at different stages of DCM, which is so far lacking in the scientific literature. In conclusion, the aims of the current study were to evaluate the effects of a single intravenous (i.v.) injection of hUC-MSCs on cardiac function and myocardial fibrosis in a male mouse model of DCM at earlier and later stages of disease progression and to determine the associated changes in key mediators of fibrosis in diabetes including dysregulation of miRNA expression and inflammation.

## Materials and methods

### Ethics statement

All animal experiments were conducted according to the recommendations in the Guide for the Care and Use of Laboratory Animals published by the US National Institutes of Health and approved by the Ethics Committee of Hebei Medical University (No.IACUC-Hebmu-2,021,035).

### Experimental animals

Male C57BL/6J mice of 6-7 weeks age were purchased from Beijing HFK Bio-Technology Co., Ltd. and were housed in a specific pathogen free (SPF) animal facility at Hebei Medical University Laboratory Animal Center. All animals were housed at constant temperature (20 ± 2^o^C) and humidity (45–55%), with a 12 h light/12 h dark cycle and free diet and water ad libitum.

### Establishment of mouse model of DM and hUC-MSC administration

**Fig. 1:**
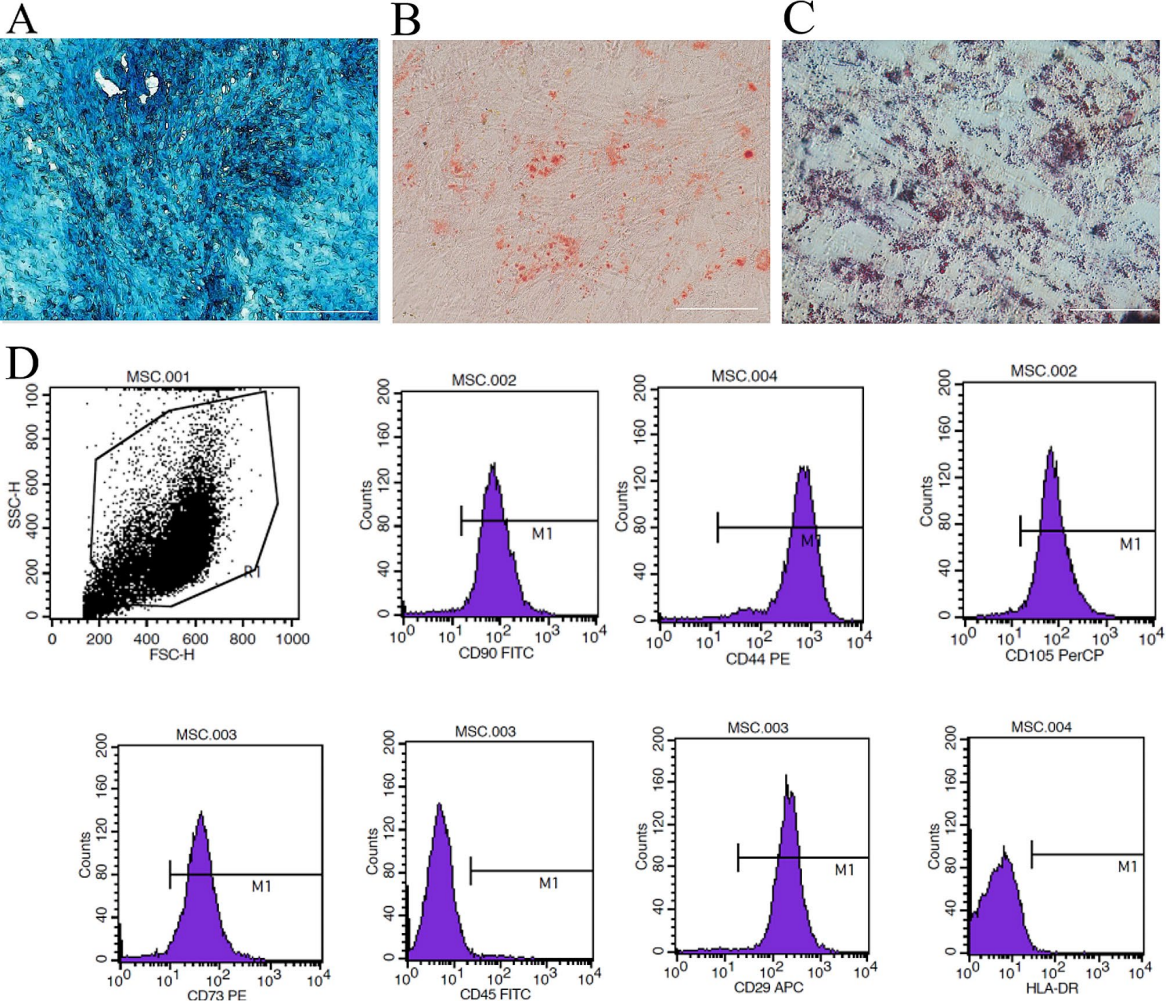
Characterization of hUC-MSCs. **A-C.** Representative photomicrographs of hUC-MSC differentiation assays for **(A)** Chrondrogenesis (Alcian blue staining). **(B)** Osteogenesis (Alizarin red staining). **(C)** Adipogenesis (Oil red O staining). Scale bar = 50 μm. Magnification, ×200. **(D)** Representative flow cytometry analysis showing hUC-MSCs forward and side scatter characteristics (dot plot) and relative fluorescence intensities following surface staining with monoclonal antibodies against CD290, CD44, CD105, CD73, CD45, CD29 and HLA-DR. All assays were repeated three times with consistent results

The groups of experimental animals described here are also the subject of a separate manuscript describing the effects of hUC-MSCs on diabetic nephropathy (10.1186/s13287-024-03647-x). A STZ model of DM with low toxicity was established as previously described [19] by intraperitoneal (i.p.) injection of STZ (Cayman Chemicals, catalog no. 18,883,664) 80 mg/kg body weight in citrate buffer pH 4.5 daily for 5 consecutive days. For each experiment, a control group of non-diabetic (Non-DM) mice was generated by 5 consecutive i.p. injections of citrate buffer alone. Blood glucose concentrations were measured once a week in all mice using a glucometer (Roche, Accu-Chek) and blood glucose test strips (Roche, Excellence). Mice with fasting blood glucose (FBG) levels ≥ 16.7 mM were considered to have DM. Two in vivo experiments were performed of 10 weeks and 18 weeks duration following induction of DM. For each, mice were randomly divided into three groups: Non-DM group, DM group (treated with i.v. injection of sterile saline 2 weeks before termination of the experiment) and DM + MSC group (treated with i.v. injection of hUC-MSCs in sterile saline 2 weeks before termination of the experiment). All study personnel were blind to treatment allocation and had no way of influencing whether an animal would receive MSCs or saline. The experimental designs are illustrated in Fig. 1A, B. For i.v. treatments, 5 × 10^5^ freshly cultured hUC-MSCs suspended in 0.2 mL of sterile saline or 0.2 mL of sterile saline alone were injected via the tail vein into mice with confirmed DM. Mice were monitored weekly for fasting blood glucose and body weight. An Animal General Distress Scoring Sheet (AGS) was completed on a daily basis with humane interventions undertaken as appropriate (see Table S1). During and after all experimental procedures, an Animal General Scoring (AGS) of mice was used to evaluate the general and traumatic pain states of the experimental animals. Throughout the experiments involving diabetes induction, cell or saline injection and post-treatment follow-up to the time of planned terminal euthanasia, the mice were monitored by a member of the research team every 1–2 days. When any of the following situations occur, we consider humanely euthanizing the animals: AGS greater than or equal to 15, weight loss of over 15% in growing mice, lack of spontaneous mobility.

### Culture, identification and characterization of hUC-MSCs

Cryopreserved hUC-MSCs were purchased from Qilu Cell Therapy Engineering Technology Co., Ltd (Shandong, China). Upon receipt, the hUC-MSCs were thawed, transferred to complete medium (DMEM, low glucose medium with 15% FBS,100 units/ml penicillin, and 100 mg/ml streptomycin) and cultured in a humidified 37 °C, 5% CO_2_ incubator. The medium was changed every 3 days. When the hUC-MSCs reached 80–90% confluence, the medium was discarded, the cell culture flask was washed three times with PBS, and the cells were lifted with MSC digestion solution (Jing Meng, Beijing, China).

To confirm the expected surface marker phenotype, freshly-lifted hUC-MSCs were stained with fluorochrome-labelled mouse anti-human antibodies against CD73, CD44, CD29, CD105, CD90, CD45, and HLA-DR and with appropriate mouse isotype control antibodies using the BD Biosciences Human MSC Analysis Kit (catalog no. 562,245, BD Biosciences, Franklin Lakes, NJ, USA) according to the manufacturer’s protocol. Flow cytometry was performed on a BD FACS Calibur (BD Biosciences) and the resulting data files were analyzed using FlowJo Software (Treestar, Ashland, OR).

To confirm tri-lineage differentiation capacity, hUC-MSCs were cultured in 6-well plates in adipogenic differentiation (Cyagen Biosciences, Guangzhou, China, catalog no. HUXUC-90,031) or osteogenic differentiation media (Cyagen Biosciences, Guangzhou, China, catalog no. HUXUC-90,021). For chondrogenic differentiation, hUC-MSCs were seeded at passage 4 in 15 mL sterile centrifuge tubes in chondrogenic differentiation medium (Cyagen Biosciences, Guangzhou, China, catalog no. HUXUC-90,041). The induction of differentiation was confirmed by standard protocols from the manufacturer for adipogenesis (Oil red O staining), osteogenesis (Alizarin red staining) and chondrogenesis (Alcian blue staining) compared to hUC-MSCs cultured under baseline conditions at the same passage number served as negative controls.

### Echocardiography

Two-dimensional, M-mode echocardiogram and tissue Doppler were performed using a Vevo 2100 ultrasound system under anesthesia with isoflurane at one day before euthanasia. In order to comprehensively evaluate the systolic and diastolic functions of the heart, the parasternal short-axis scan and the apical four-chamber scan were selected for assessment. Using the parasternal short-axis scan, the following parameters were recorded: heart rate (HR), left ventricular internal diameter at end-diastole (LVIDd), left ventricular internal diameter at end-systole (LVIDs), left ventricular anterior wall thickness at end-diastole (LVAWd), left ventricular anterior wall thickness at end-systole (LVAWs), left ventricular posterior wall thickness at end-diastole (LVPWd), left ventricular posterior wall thickness at end-systole (LVPWs), left ventricular weight (LVW), stroke volume (SV), cardiac output (CO), left ventricular ejection fraction (LVEF) and fractional shortening (FS). The first six indicators measured by ultrasound images can reflect the shape of the heart at the end of systole or diastole. The remaining indicators were calculated based on the first six indicators. LVEF and FS are the most typical indicators to evaluate cardiac systolic function [20]. By the apical four-chamber scan, we measured the ratio of peak early diastolic filling velocity to late atrial filling velocity (*E*/*A*) and isovolumic relaxation time (IVRT) which mainly reflect the diastolic function of the heart [21]. In the case of the echocardiography experiments, the procedure was performed by an expert technologist assisted by a member of the research team (LBX). The animals were anaesthetized with isoflurane gas throughout the procedure. The mouse was placed supine on the procedure table, with its limbs fixed on the table. The mouse’s mouth and nose were connected to a plastic tube for continuous gas anesthesia. After the mouse’s heartbeat stabilizes, echocardiogram data was recorded by the technologist. The physiological state of the mouse was continuously monitored during operation and the rate of gas flow is adjusted as necessary. The animals were observed in cages for 20 min following echocardiography to ensure adequate recovery from anesthesia before being returned to the housing facility. The cardiac ultrasound measurements were performed in triplicate on each animal and the mean values were calculated. The statistical analysis for each parameter was performed using mean values of six mice in each group.

### Tissue harvest, histopathology staining and image analysis

Following humane euthanasia, mouse hearts were isolated, weighed, fixed in paraformaldehyde and then embedded in paraffin. For each paraffin-embedded heart, whole-organ 4-µm thick sections were prepared using a microtome. For initial assessment of tissue quality and structure, a single heart section from one animal per group was stained with hematoxylin and eosin (H&E) and inspected by light microscopy. Next, for each experiment, whole heart sections from 3 different levels of the organ were stained with Masson’s trichrome (MT) for 6 mice per group and three sections were randomly selected for each level. The protocols used for H&E and MT staining are provided in Supplemental Methods. For image analysis of MT-stained sections, 6 fields from each section were selected for the interstitial area, and 3 fields were selected for the perivascular area. Images were collected at 200×magnification and the MT-stained sections were analyzed in blinded fashion using a light microscope (Olympus BX53, Japan). Quantitative image analysis was performed using Image-Pro Plus 6.0 software (Image-pro Plus, Media Cybernetics, Inc., USA). For quantification of interstitial fibrosis, collagen volume fraction (CVF) was calculated for each field as the ratio of the blue-stained area to the total interstitial area. For quantification of perivascular fibrosis, collagen vascular fraction, defined as the ratio of the blue-stained perivascular collagen area to the total luminal area (PVCA/LA), was calculated for each vessel within the field [22]. In total 90 fields per heart were analyzed for interstitial fibrosis and 45 fields per heart were analyzed for perivascular fibrosis. The final CVF and PVCA/LA values, expressed as %, were derived for each heart from the average values of all analyzed fields. The analyses in each field were conducted by three independent researchers (HJJ, FBF and CYM), and subsequently, the averages were calculated and subjected to statistical analysis by another researcher (LBX).

### Selection of candidate differentially expressed miRNAs and mRNAs from publically available sources

In order to identify miRNAs with altered expression in diabetic mouse heart, we selected Non-DM and DM group sequencing data from a miRNA high-throughput sequencing (series GSE210036 [23]) and generated a heatmap of the top 50 differentially-expressed miRNAs. From this, we selected six fibrosis-associated miRNAs: let-7f, miR-26a, miR-29a, miR-29b, miR-29c and miR-133a. Based on the recent review by Z-Q Jin [24] we also selected three additional candidate miRNAs: miR-34a, miR-155 and miR-326. The relative expression of these nine miRNAs, and subsequently, the relative expression of candidate mRNAs in heart tissue from the experimental groups of the current study were then determined by qRT-PCR (methods described below).

To identify candidate mRNAs with altered expression in the heart in diabetes, we also selected publically available Non-DM and DM group data from a recently performed mRNA high-throughput sequencing analysis (series GSE161052 [25]) and generated a volcano plot with annotation of the top 18 differentially expressed mRNAs. We noted that these mRNAs included transcripts encoding the alpha 1 chains of collagen I and collagen III and selected these mRNAs for qRT-PCR analysis in hearts from the current study due to their relevance to fibrosis and prior identification of COL1A1 as a target for miRNA-133a. In addition, 6 other fibrosis-related mRNAs (α-SMA, TGF-β, Smad2, Smad3, Smad4 and FGF1) potentially regulated by miRNA-133a were also selected for quantitative analysis in heart tissue from our experimental groups.

### RNA extractions and quantitative reverse transcription and polymerase chain reaction (qRT-PCR) analyses

After euthanasia, one-half of the fresh heart tissue was stored in a -80 ° C freezer for further use (*n* = 6 per group). For qRT-PCR analyses, total miRNA or mRNA were extracted from heart tissue using miRNA extraction and isolation kit (DP501, TIANGEN, Beijing) and mRNA extraction and isolation kit (DP424, TIANGEN, Beijing) respectively according to the manufacturers’ instructions. The miRNA and mRNA preparations were transcribed to cDNA by miRNA First Strand cDNA Synthesis (Tailing Reaction, B532451, Sango Biotech, Shanghai) and cDNA first strand synthesis kit (ZS-M14003, ZHONGSHI TONTAU, Tianjin), respectively according to the manufacturers’ instructions. Quantitative PCR was performed with a SYBR Green PCR master mix (ZS-M13010, ZHONGSHI TONTAU, Tianjin). Because miRNA was generally composed of more than 20 bases, different from general mRNA, we adopted the poly(A) tailing method for reverse transcription of miRNA. The tailed reverse transcription assay utilizes poly (A) polymerase for mature miRNA plus poly (A) tail, followed by 5'- end universal tagged oligo dt as the reverse transcription primer to obtain the human elongated miRNA cDNA first strand, and finally fluorescence quantitative PCR detection with a reverse primer complementary to the universal tag sequence by dye method or probe method. Universal PCR primer R (B661601-0002, Sango Biotech, Shanghai) were the same for all miRNAs and internal control U6. The primer F sequences of miRNA are listed in Table S2 and primer sequences of mRNA are listed in Supplemental Table S3. A melt curve was included to ensure primer specificity. Experiments were performed in triplicate, and results were normalized to U6 or β-actin expression (2^−ΔΔCT^ method). Each qRT-PCR reaction was performed in triplicate and the average results for the three technical replicates were used for statistical analyses.

### Immunohistochemical experimental results analysis

All tissues were fixed in 4% paraformaldehyde overnight and embedded in paraffin. For IHC staining [26], 4µM sections were prepared and were deparaffinized and then treated with an antigen-retrieval solution at 98^o^C for 25 min followed by a peroxidase-blocking solution (Dako) for 15 min, and then with a streptavidin-biotin blocking solution for 1 h. Next, the sections were incubated for 2 h at room temperature with one of the following antibodies: rabbit anti-mouse CD3 mAb (1:200, Servicebio, China); rabbit anti-mouse F4/80 mAb (1:200, Servicebio, China). Following this, the sections were washed and incubated with biotinylated goat anti-rabbit secondary antibodies (1:500, Servicebio, China) for 2 h at room temperature. The sections were then dehydrated, mounted and imaged using an Olympus BX53 light microscope. Quantitative image analysis was performed using Image-Pro Plus 6.0 software (Image-pro Plus, Media Cybernetics, Inc., USA). For quantification, the antibody-positive (brown-stained) area was calculated for each field and expressed as the ratio of the brown-stained area to the total area [27]. Three sections were analyzed for each tissue sample from *n* = 6 mice per group. For each section, 6 fields were randomly selected for analysis (total 18 fields per heart). The average of the final % positive area values the 18 fields were derived for each heart. The analysis was conducted independently by three researchers (LBX, FBF and CYM), and the average values of the three independent analyses were used by another researcher (HJJ) for the final statistical analysis.

### Preparation of serum and tissue samples and performance of enzyme-linked immunosorbent assay (ELISA)

Mice were fasted for 6 h then anesthetized by inhaling 2–3% isoflurane. Blood samples were collected by abdominal aortic puncture into clotting tubes and left at room temperature for 30 min. Once clots had formed, the blood samples were centrifuged at 1200 xg at 4 °C for 15 min to separate serum. The serum fraction was carefully withdrawn from each sample by pipetting and was divided into 200 µL aliquots in sterile tubes and stored at -80 °C until used for biochemical and cytokine assays. Following humane euthanasia and tissue procurement, pieces of fresh heart tissue were placed in sterile tubes and stored immediately at -80 °C until use for tissue homogenate preparation for EILSA. The heart tissue was cut into about 500 mg slices on ice, then placed in 2mL ice bath pre-cooled phosphate buffer salt ice bath ground, then diluted to 5 mL with the same solution. The samples were then centrifuged at 1500 xg for 15 min. The clarified supernatant was divided into 1mL aliquots and stored at -80 °C for future use. Serum concentrations of interleukin-1β (IL-1β), interleukin (IL-6) tumor necrosis factor (TNF), transforming factor beta 1 (TGFβ1) and fibroblast growth factor 1 (FGF-1) were quantified using ELISA kits (abclonal, Wuhan, China) according the manufacturer’s instructions. The concentration of IL-6 was also quantified in heart tissue homogenates by the same protocol. For all ELISAs, each sample was analyzed in triplicate and the mean values for *n* = 6 animals per group were used for statistical analyses.

### Statistical analysis

For each experimental procedure, the researchers performing analyses remained blinded to group assignment until all analyses were complete, following which the data were unblinded and statistical analyses were performed. For all experiments, group data were expressed as mean ± SD and analyzed using GraphPad Prism 6. The differences between the groups were compared using ordinary one-way analysis of variance (ANOVA) or multiple comparisons of 2-way ANOVA. PP < 0.05 was considered statistically significant. For bioinformatics analyses of publicly available miRNA and mRNA datasets, R programming language (version 4.1.3) and R studio (version 3.3.0) were used.

## Results

### Characterization of human umbilical cord-derived mesenchymal stromal cells

**Fig. 2:**
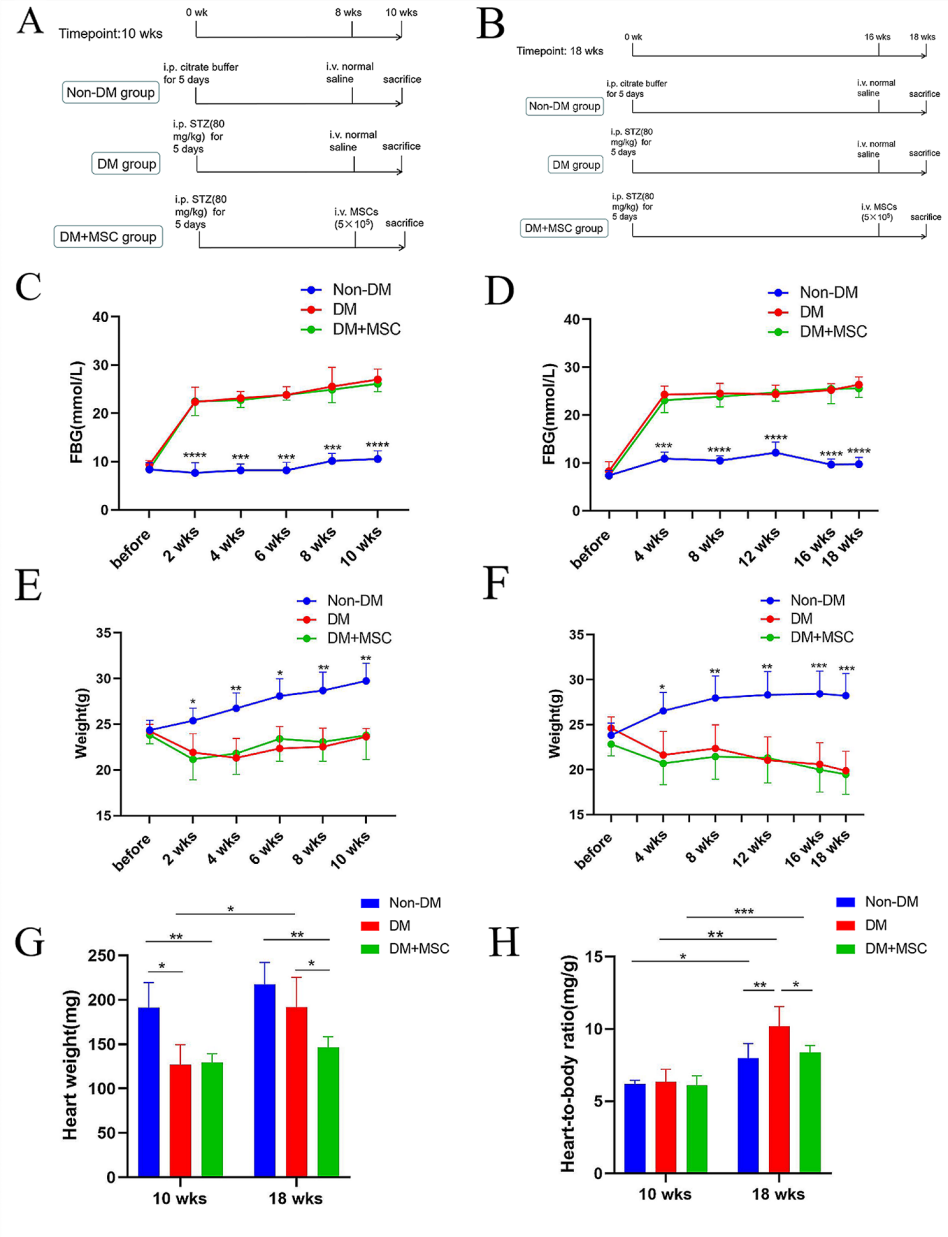
Design, monitoring and terminal heart weight analysis of mouse diabetes model experiments (**A-B**). Summaries of experimental groups and time points for two mouse diabetes model experiments involving i.v. injections of saline or hUC-MSCs at earlier (**A**) and later (**B**) time-points following induction of diabetes. **C-D.** Graphs of baseline (before) and 2-weekly fasting blood glucose concentrations (in mmol/L) of three groups of mice each from experiments of 10 weeks (**C**) and 18 weeks (**D**) duration. **E-F.** Graphs of baseline (before) and 2-weekly body weights (in g) in the same experimental groups. **G-H.** Graphs of heart weights (**G**, in mg) and heart-body weight ratios (**H**, in mg/g) at the end-points of experiments of 10 weeks and 18 weeks duration *n* = 6 per group for all experiments. Results presented as mean ± SD. Statistical analysis was performed by 2-way Analysis of Variance (ANOVA) with adjustment for multiple comparisons. *:*P* < 0.05; **:*P* < 0.01; ***:*P* < 0.001; ****:*P* < 0.0001. Abbreviations: Non-DM = non-diabetic group; DM = diabetes mellitus with injection of saline; DM + MSC = diabetes mellitus with injection of hUC-MSCs, i.p.– intra-peritoneal, i.v. = intravenous

For validation of hUC-MSC cultures used for the in vivo studies, it was first determined whether the cells met the International Society for Cellular Therapy (ISCT) minimal criteria. The cells demonstrated an adherent growth pattern, were highly proliferative within serum-containing medium (data not shown), exhibited multi-lineage differentiation in assays of adipogenesis, osteogenesis and chondrogenesis (Fig. 2A-C), and demonstrated the expected positive and negative staining ratios of related surface markers in flow cytometry analyses [CD29 (98.91%), CD44 (99.43%), CD73 (96.78%), CD90 (98.22%), CD105 (98.56%), CD45 (0.88%), HLA-DR (0.5%)] (Fig. 2D).

### Establishment of male mouse model of diabetes mellitus with systemic hUC-MSC administration

Groups of mice with DM, along with Non-DM control groups, were established by intraperitoneal injection of streptozotocin (STZ) or citrate buffer respectively and were used for two, 3-group in vivo experiments involving i.v. injection of hUC-MSCs or vehicle (sterile saline) at earlier (8 weeks) and later (16 weeks) time-points followed by 2 week observation periods before euthanasia (Fig. 2A, B). As shown in Fig. 2C-F, the fasting blood glucose (FBG) of both groups of DM mice increased significantly while the body weights decreased throughout the time courses of the two experiments compared to the Non-DM groups. Notably, for both experiments, there were no differences between the DM and DM + MSC groups in FBG or body weight (Fig. 2C-F). These results confirmed establishment of a model of DM for up to 18 weeks and indicated that i.v. administration of single doses of hUC-MSCs at 8 and 16 weeks following induction of DM did not ameliorate hyperglycemia or weight loss.

### Effects of early or later hUC-MSC administration on heart weights of diabetic animals

At the 10 week time-point (following hUC-MSC injection at 8 weeks), the heart weights of mice in the DM group and the DM + MSC groups were significantly less than those of the Non-DM group (Fig. 2G), while heart-to-body weight ratio did not differ among the three groups (Fig. 2F). For this earlier time of administration, no difference in heart weight was seen between DM and DM + MSC groups. At the 18 week time-point (following hUC-MSC injection at 16 weeks), the heart weight of mice in the DM group was not different to those of the Non-DM group and the heart-to-body weight ratio was higher (Fig. 2G, H). In contrast, for this later time-point of hUC-MSC injection, the heart weights of the DM + MSC group remained significantly lower than those of the Non-DM group and the heart-to-body weight remained similar (Fig. 2G, H). Comparing results across the two time-points for the DM group, both heart weight and heart-to-body weight ratio increased substantially between 10 and 18 weeks and these trends were largely prevented by hUC-MSC administration (Fig. 2G, H). Thus, while early administration of hUC-MSCs did not prevent the loss of heart weight following 10 weeks of DM, the progressive increase in heart weight and heart-to-body-weight ratio that occurred between 10 and 18 weeks after DM onset was prevented by a later injection of hUC-MSCs.

### Effects of early or later hUC-MSC administration on heart function of diabetic animals

**Fig. 3:**
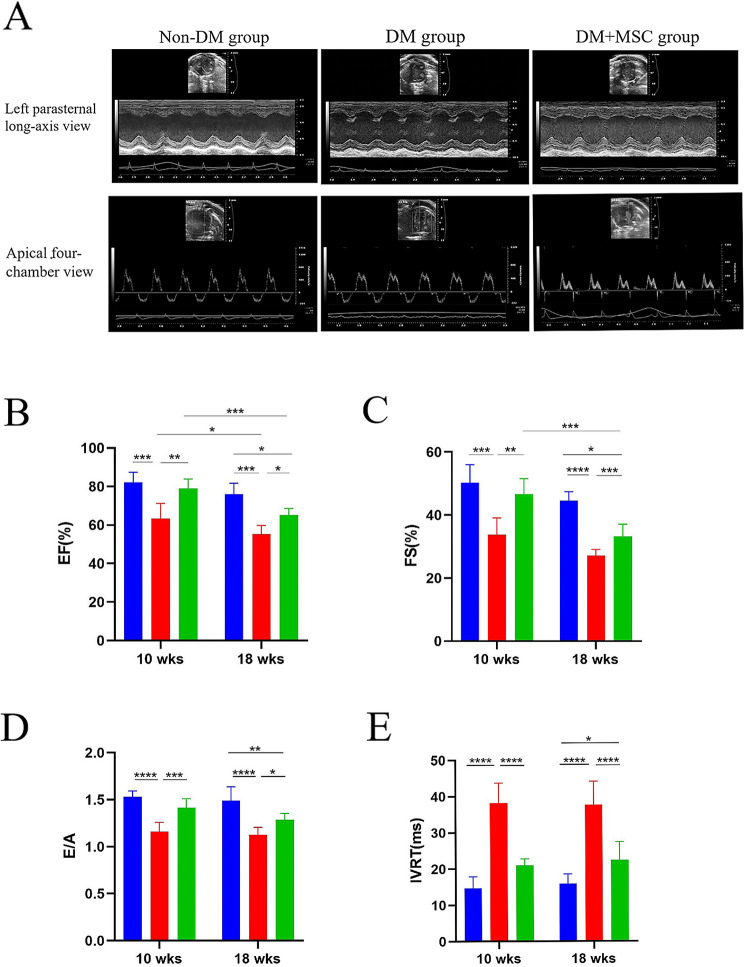
Echocardiographic analysis of systolic and diastolic heart function of mouse diabetes model experiments. **A.** Representative examples of 2D echocardiography images from the left parasternal long-axis view (upper) and the apical four-chamber view (lower) in three groups of experimental mice. **B-E.** Graphs of echocardiographic indices of systolic function - EF (**B**), FS (**C**); and diastolic function - *E/A* (**D**) and IVRT (**E**) in three groups of animals each from experiments of 10 weeks and 18 weeks duration. *n* = 6 per group for all experiments. Results presented as mean ± SD. Statistical analysis was performed by 2-way Analysis of Variance (ANOVA) with adjustment for multiple comparisons. *:*P* < 0.05; **:*P* < 0.01; ***:*P* < 0.001; ****:*P* < 0.0001. Abbreviations: Non-DM = non-diabetic group; DM = diabetes mellitus with injection of saline; DM + MSC = diabetes mellitus with injection of hUC-MSCs; EF = ejection fraction; FS = fractional shortening; E/A = E/A ratio; IVRT = isovolumic relaxation time

Echocardiography was used to evaluate the effects of DM and i.v. hUC-MSC administration on cardiac function. Measurements were taken via the parasternal short-axis and the apical four-chamber views (Fig. 3A). Among the measured indicators, left ventricular ejection fraction (LVEF) and fractional shortening (FS) reflected systolic function (Fig. 3B, C), while E/A ratio and isovolumetric relaxation time (IVRT) mainly reflected diastolic function (Fig. 3D, E). As shown, in comparison to the Non-DM group, mice in the DM group developed cardiac systolic (reduced EF and FS) and diastolic (reduced *E/A* ratio and ICRT) dysfunction as early as 10 weeks after STZ injection. The systolic dysfunction worsened between 10 and 18 weeks. For both time-points, hUC-MSC administration was associated with significant improvements in systolic and diastolic function compared to control DM groups. Results for other echocardiographic parameters are summarized in Table 1. The echocardiographic studies provided evidence for progressive cardiac dysfunction in the mouse model of DM and for beneficial effects of single i.v. injections of hUC-MSCs following both shorter and longer DM duration.

**Table 1:** Summary of results of cardiac functional indices from echocardiography of three groups of mice each from experiments of 10 weeks and 18 weeks duration

	Non-DM group	DM group	DM + MSC group
Heart Rate(bpm)			
10 wks	501.6 ± 44.3	380.6 ± 50.4 *	389.3 ± 44.4 *
18 wks	515.7 ± 47.1	375.8 ± 55.8 *	382.6 ± 66 *
LVIDd(mm)			
10 wks	3.6 ± 0.4	4.0 ± 0.2 *	3.6 ± 0.3
18 wks	3.7 ± 0.3	4.1 ± 0.4 *	3.6 ± 0.2 #
LVIDs(mm)			
10 wks	2.7 ± 0.3	3.2 ± 0.3 *	2.6 ± 0.3 #
18 wks	2.6 ± 0.3	3.2 ± 0.4 *	2.6 ± 0.4
SV(µL)			
10 wks	40.1 ± 4.4	30.9 ± 1.9 *	36.2 ± 2.8 *#
18 wks	39.4 ± 5.5	29.4 ± 2 *	35 ± 3.4 *
CO(mL/min)			
10 wks	19.8 ± 2.2	13.7 ± 3.1 *	14.4 ± 2.9 *
18 wks	20.2 ± 2.7	12.5 ± 1.4 *	14 ± 2.8 *
LVAWd(mm)			
10 wks	0.9 ± 0.1	0.7 ± 0.1 *	0.7 ± 0.1 *
18 wks	0.9 ± 0.1	0.8 ± 0.1	0.7 ± 0.1 *
LVAWs(mm)			
10 wks	1.3 ± 0.1	1 ± 0.1 *	1 ± 0.1 *
18 wks	1.4 ± 0.1	1.1 ± 0.1 *	1 ± 0.1 *
LVPWd(mm)			
10 wks	0.8 ± 0.1	0.7 ± 0.2	0.7 ± 0.1
18 wks	0.8 ± 0.1	0.7 ± 0.1	0.7 ± 0.1
LVPWs(mm)			
10 wks	1.2 ± 0.1	1 ± 0.1 *	1 ± 0.1 *
18 wks	1.2 ± 0.1	1 ± 0.1 *	1 ± 0.1 *
LVW(mg)			
10 wks	84.7 ± 7.4	67.1 ± 8.2 *	66.9 ± 5.8 *
18 wks	93.5 ± 8.5	76.6 ± 5 *	70.4 ± 10 *#

*Abbreviations* Non-DM = non-diabetic group; DM = diabetes mellitus with injection of saline; DM + MSC = diabetes mellitus with injection of hUC-MSCs; HR = heart rate; LVIDd = left ventricular internal diameter at end-diastole; LVIDs = left ventricular internal diameter at end-systole; SV = stroke volume; CO = cardiac output; LVAWd = left ventricular anterior wall thickness at end-diastole; LVAWs = left ventricular anterior wall thickness at end-systole; LVPWd = left ventricular posterior wall thickness at end-diastole; LVPWs = left ventricular posterior wall thickness at end-systole; LVW = left ventricular weight. All values are expressed as means ± SD. *n* = 6 per group. * = significant (*p* < 0.05) compared to Non-DM group, # = significant (*p* < 0.05) compared to DM group. Data were analyzed using one-way ANOVA followed by Tukey’s post hoc test for multiple comparisons

### Effects of early or later hUC-MSC administration on myocardial structure of diabetic animals

**Fig. 4:**
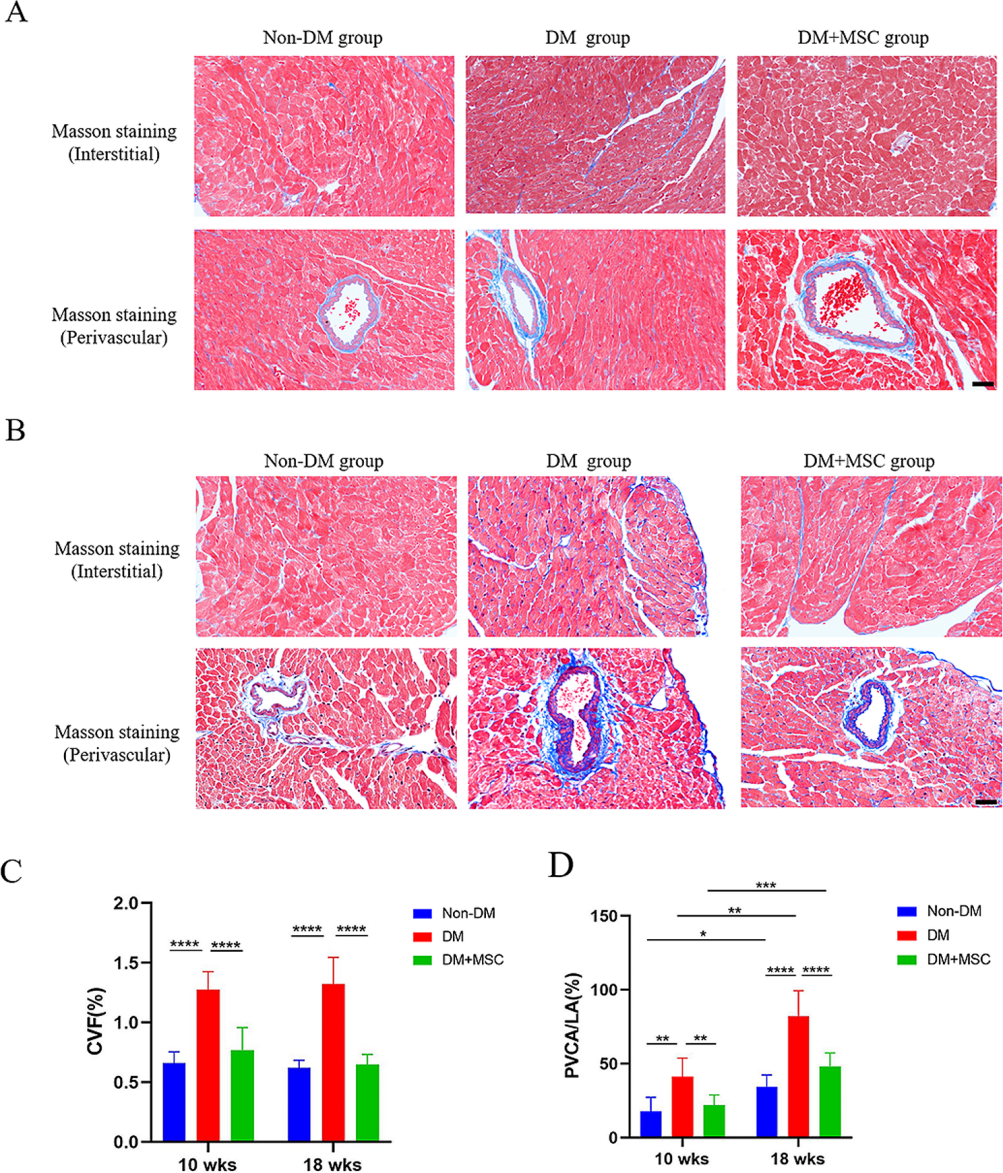
Histological analysis of cardiac fibrosis in mouse diabetes model experiments. **A-B.** Representative photomicrographs of cardiac tissue sections stained with Masson’s trichrome illustrating interstitial and perivascular fibrosis in three groups of animals each from diabetes model experiments of 10 weeks (**A**) and 18 weeks (**B**) duration. Scale bar = 20 μm. Magnification, 200×. **C-D.** Graphs of quantitative image analyses of interstitial fibrosis (**C**, CVF %) and perivascular fibrosis (**D**, PVCA/LA %) performed of Masson’s trichrome-stained cardiac tissue sections from the same experimental groups. Results presented as mean ± SD. Statistical analysis was performed by 2-way Analysis of Variance (ANOVA) with adjustment for multiple comparisons. *:*P* < 0.05; **:*P* < 0.01; ***:*P* < 0.001; ****:*P* < 0.0001. Abbreviations: Non-DM = non-diabetic group; DM = diabetes mellitus with injection of saline; DM + MSC = diabetes mellitus with injection of hUC-MSCs; CVF (%) = collagen volume fraction; PVCA/LA (%) = perivascular collagen volume/luminal area

H&E staining of cardiac tissue sections revealed some disorganized patterning of cardiomyocytes in the left ventricles of DM and DM + MSC groups at both time-points compared to Non-DM hearts (Fig S1.). For more quantitative assessment of myocardial structure, the extent of interstitial and perivascular fibrosis was compared among the groups by Masson’s trichrome staining and image analysis of hearts collected at 10 weeks (Fig. 4A, C) and 18 weeks (Fig. 4B, D) after induction of DM. As shown in Fig. 4C and D, DM was associated with significantly increased % collagen vascular fraction (CVF) in the myocardial interstitium and increased % perivascular collagen area/luminal area (PVCA/LA) compared to Non-DM. Perivascular fibrosis increased in all 3 groups between 10 weeks and 18 weeks but the increase was more marked in the DM group. For both earlier and later time-points, hUC-MSC administration resulted in prevention of the DM-associated intra-cardiac fibrotic changes. To evaluate overall cardiac vascularity, arteriole density was counted in H&E stained heart sections. As shown in Fig. S1B arteriole density was reduced in DM compared to Non-DM groups at both time-points but this was not reversed in the DM-MSC groups.

### Effects of early or later hUC-MSC administration on cardiac expression of miRNA and fibrosis-related mRNA in diabetic animals

**Fig. 5:**
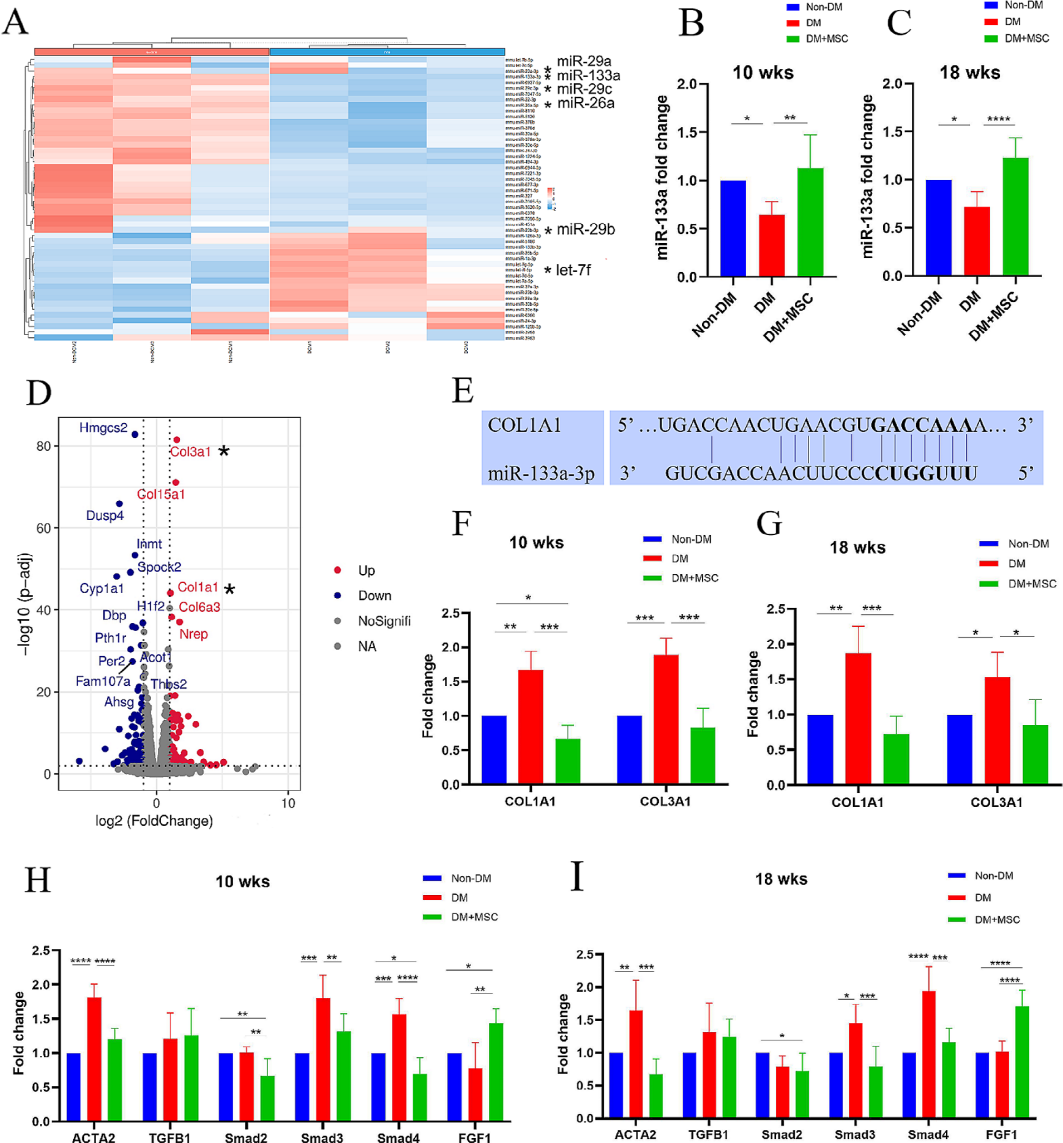
Analyses of micro RNAs and messenger RNAs in cardiac tissue from diabetes model experiments: **A.** Heatmap, derived from publically available dataset GSE210036 of the top 50 differentially expressed miRNAs in hearts of mice with diabetic cardiomyopathy (DCM) to those of mice without DCM (Non-DCM). **B-C.** Graphs of quantitative RT-PCR analyses of the relative expression of microRNA-133a in heart tissue of three groups of mice each from experiments of 10 weeks (**B**) and 18 weeks (**C**) duration. Levels for DM and DM + MSC groups are expressed as fold change relative to the Non-DM group. **D**. Volcano plot derived from publically-available dataset GSE161052 of differentially expressed mRNAs in hearts of mice with DM compared to those without DM. Annotations indicate the gene names for the top 18 differentially expressed mRNAs (red = higher in DM, blue = lower in DM). * = miRNAs and mRNAs selected for validation in the current study. **E.** The binding site of Col1A1 and miR-133a from website TargetScan. **F-G**. Graphs of quantitative RT-PCR analyses of the relative expression of mRNAs COL1A1 and COL3A1 in heart tissue of three groups of mice each from experiments of 10 weeks (**F**) and 18 weeks (**G**) duration. Levels for DM and DM + MSC groups are expressed as fold change relative to the Non-DM group. **H-I.** Graphs of quantitative RT-PCR analyses of the relative expression of six selected mRNAs related to fibrosis in heart tissue of three groups of mice each from experiments of 10 weeks (**H**) and 18 weeks (**L**) duration. Levels for DM and DM + MSC groups are expressed as fold change relative to the Non-DM group. For B, C, F, G, H, and G, all values are expressed as mean ± SD of *n* = 6 biological replicates. Statistical analysis was performed by one-way ANOVA. *P < 0.05, **P < 0.01, ***P < 0.001, ****P < 0.0001, respectively. Abbreviations: Non-DM = non-diabetic group; DM = diabetes mellitus with injection of saline; DM + MSC = diabetes mellitus with injection of hUC-MSCs

To identify potential candidates for DM-associated, myocardial miRNAs that may be modulated by hUC-MSC administration, we first generated a heatmap (Fig. 5A) of the top 50 differentially expressed miRNAs in publically available datasets for cardiac tissue of groups of male C57BL/6 mice with and without STZ-induced DM for 12 weeks from the recently-published study of Cagnin et al. (GSE210036 [23]),. From these, 6 miRNAs were selected for quantitative (q)RT-PCR analysis in heart tissue samples from our two in vivo experiments. An additional 3 miRNAs, selected from the recent review of Z-Q Jin were also quantified [28]. The results of qRT-PCR analysis for these 9 candidate miRNAs are shown in Fig. 5B, C and Fig S2. As shown in Fig. 5B, C myocardial expression of miRNA-133a, an anti-fibrotic miRNA, was reduced in DM compared to Non-DM mice at 10 weeks and 18 weeks but was unchanged in DM-MSC groups at both time-points. For the other eight miRNAs that were studied, none were significantly different between DM and Non-DM groups at either time point (Fig S2).

To screen for mRNAs that have been found to be upregulated within the heart in DM and may be regulated by miRNA-133a, we generated a volcano plot (Fig. 5D) of the differentially expressed transcripts (DM vs. Non-DM) from the publically-available high-throughput mRNA sequencing dataset of Wei et al. (GSE161052 [25]). Among the upregulated transcripts in this analysis, Col1A1 has been shown by Castoldi et al. to be a direct target of miRNA-133a [29]. According to the relevant information in the Targetscan database, we found the possible binding sites of miRNA-133a and Col1A1 (Fig. 5E). We proceeded to perform qRT-PCR of heart tissue samples from our two in vivo experiments to quantify mRNAs for Col1A1 and Col3A1 (Fig. 5F, G). These analyses showed that the two collagen gene transcripts were increased in hearts of DM compared to Non-DM mice at 10 and 18 weeks and that hUC-MSC administration at both earlier and later time-points had prevented the upregulation.

Next, the mRNA expression of other pro-fibrotic gene products - ACTA2(α-SMA), TGFB1(TGF-β), Smad2, Smad3, Smad4 - and the anti-fibrotic mRNA FGF1 was determined in the 10 week and 18 week cardiac tissue samples by qRT-PCR (Fig. 5I, J). As shown, myocardial expression of mRNAs encoding ACTA2, Smad3, and Smad4, was increased in DM compared to Non-DM animals at both time-points. In all cases, hUC-MSC administration was associated with reversal of the change. Strikingly, myocardial expression of mRNA for FGF-1, which has been reported to inhibit fibroblast activation, collagen production and pro-fibrotic TGF-β1 signaling [30], was increased in DM + MSC groups compared to both DM and Non-DM groups (Fig. 5I, J).

It was concluded that the beneficial effects on myocardial function and structure of single i.v. injections of hUC-MSCs at earlier and later time-points following induction of DM are accompanied by preservation of the expression of the anti-fibrotic miRNA, miR-133a, prevention of the up-regulation of pro-fibrotic mRNAs and increased expression of the anti-fibrotic factor FGF-1.

### Effects of early or later hUC-MSC administration on inflammation-related mRNAs within heart tissue and on systemic levels of inflammation- and fibrosis-related cytokines in diabetic animals

**Fig. 6:**
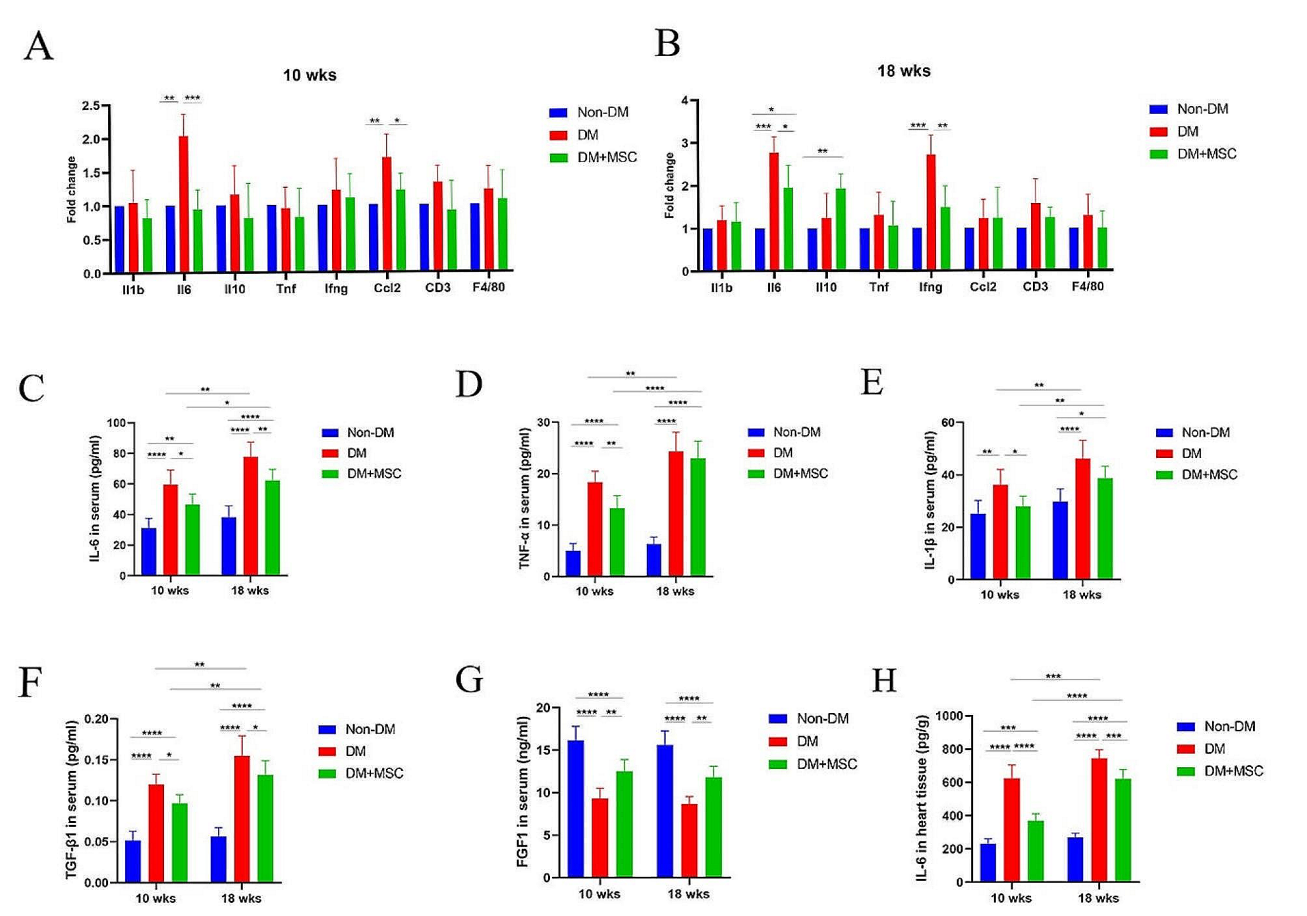
Analyses of Inflammatory factors and T cell and macrophage markers related messenger RNAs and pro-inflammatory cytokines ELISA results in serum and cardiac tissue. **A**-**B.** hUC-MSCs treatment regulated the expression of inflammation related mRNAs. **C**-**G**. Serum concentrations of IL-6 (**C**) and TNF-α (**D**) and IL-1β (**E**) and TGFβ1(**F**) and FGF1(**G**) were measured using ELISA kits. **H.** The concentrations of IL-6 in heart tissue homogenate. All values were expressed as mean ± SD in triplicate (*n* = 6), each sample had three multiple holes, and the average value is taken “*”, “**”, “***”, indicate significance levels at *P* < 0.05, *P* < 0.01, *P* < 0.001, respectively. Statistical analysis was performed using Multiple comparisons of 2-way ANOVA

To determine whether the protective effect of hUC-MSC injection was associated with modulation of intra-myocardial inflammation in DCM, the relative expression levels of mRNAs encoding cytokines/chemokines (IL-1β, IL-6, IL-10, TNF, IFNγ, CCL2), a T cell-specific marker (CD3) and a macrophage-specific marker (F4/80) were quantified in heart tissue samples from all groups. As shown in Fig. 6A, B, the expression of mRNA encoding IL-6 was significantly increased in heart tissue of the DM group compared to Non-DM and DM-MSC groups at both time-points. Expression of mRNA encoding CCL2 was also higher in the DM group compared to the other two groups at 10 weeks but not at 18 weeks. At 18 weeks, mRNA for IFNγ was increased in hearts of the DM group compared to Non-DM and DM-MSC groups, while that for the anti-inflammatory cytokine IL-10 was increased in the DM-MSC group. The mRNA expression diferences for IL-6 at both time-points were confirmed at protein level by ELISA of tissue homogenates (Fig. 6H). No significant expression changes were observed across the groups for CD3 and F4/80 mRNAs. Furthermore, IHC staining of cardiac tissue for CD3 and F4/80 (Fig S3 A-D) did not reveal detectable T cell or macrophage infiltrates in samples from diabetic animals at either time-point. It was concluded that, in this model of DCM, cardiac dysfunction and cardiac fibrosis were accompanied at both time-points by relatively modest increases in the intra-cardiac expression of specific inflammatory mediators which was modulated by hUC-MSC injection. The cardiac abnormalities observed were not, however, associated with an overt cellular inflammatory infiltrate.

In regard to systemic inflammation, the serum concentrations of all IL-1β, IL-6 and TNF were higher in the DM group compared to the Non-DM group at 10 weeks and were increased further at 18 weeks (Fig. 6C, D, E). At 10 weeks, serum levels of all 3 pro-inflammatory cytokines were lower in the DM-MSC compared to DM group while, at 18 weeks, only IL-6 remained significantly lower in the DM-MSC group. Notably, serum concentrations of the pro-fibrotic cytokine TGFβ1 showed similar between-group differences to IL-6 at both time-points while serum levels of the anti-fibrotic cytokine FGF-1 were lower in the DM compared to the Non-DM group and were partially restored in the DM-MSC group (Fig. 6C, D, E). These results confirmed that DM was associated with a progressive, systemic pro-inflammatory state that was more potently suppressed by hUC-MSC injection at an early compared to later time-point. At both time-points, DM-induced systemic inflammation occurred concomitantly with a hUC-MSC-responsive pro-fibrotic profile.

## Discussion

Diabetic cardiomyopathy is a serious complication of diabetes mellitus, which leads to heart failure and increased mortality [31]. Cardiac structural abnormalities are the main pathological changes in DCM, including cardiac fibrosis and cardiac hypertrophy, and these pathological alterations contribute to the development of heart failure symptoms [32–34]. The current clinical treatment of DCM focuses predominantly on relieving these symptoms and, while there has been much progress in pharmacological approaches to the treatment of heart failure, there is no effective method to prevent the progression of myocardial fibrosis in DCM [35]. Due to their paracrine properties and readily accessible source material, hUC-MSCs have been identified as a promising, novel treatment for DCM [36]. Potential mechanisms for their therapeutic benefits include paracrine-mediated immunomodulatory, pro-angiogenic and anti-inflammatory effects [37]. It is worth noting that MSCs have been reported to exhibit both antidiabetic properties and cardioprotective properties, which, in combination, could be highly beneficial for the prevention or reversal of DCM [38]. In keeping with this, intravenously delivered MSCs have been demonstrated to alleviate cardiac inflammation in rodent models of diabetes [39].

In this study, we used tail vein injection of hUC-MSCs to observe whether the cardiac dysfunction and pathological changes of fibrosis seen in diabetic male mice would be improved. Male mice were chosen for this project to avoid experimental variability related to female hormonal effects on insulin sensitivity and to exploit the higher rate of development of STZ-induced diabetes in male compared to female mice [40, 41]. However, using only male mice limited the generalizability of the results and conclusions. The experiments and conclusions in this study only support the efficacy of hUC-MSCs in treating male C57BL/6J mice. At the same time, the expression changes of mRNA and miRNA were analyzed by RT-qPCR in order to explore the therapeutic target of hUC-MSC administration. To gain more insight into the influence of diabetes duration on the effects of systemic hUC-MSCs on DM and DCM, we performed experiments in which the intervention was made at earlier and later stages of disease. Of interest, even at the earlier time-point, we observed no evidence of an anti-diabetic effect of i.v. hUC-MSCs– likely reflecting the fact that DM due to islet destruction by STZ had been established for 8 weeks or more before cells were administered. For the purpose of interpreting our results, this indicates that the observed improvements in cardiac function and structure following hUC-MSC infusion likely reflect direct effects on DCM pathophysiology rather than indirect benefits due to reduction in hyperglycemia. Our application of echocardiography provided a comprehensive non-invasive evaluation of heart function and has been used by others to demonstrate beneficial effects of therapeutic interventions in models of DCM [42]. It also provides a means to compare cardiac functional abnormalities observed in the mouse model, with those reported for human subjects with DCM, in whom diastolic dysfunction (reflected by E/A and IVRT) may be more prevalent and occur earlier then systolic dysfunction [16]. In keeping with this, there was marked increase in IVRT by 10 weeks after the induction of DM in our experiments which persisted at 18 weeks and was largely prevented by hUC-MSC injection. Nonetheless, evidence of systolic dysfunction and of its amelioration by hUC-MSCs was also observed by echocardiography at both time-points. It is important, however, to consider to what extent the functional findings from a mouse model of DCM are applicable to cardiac dysfunction in humans. For example, the reported left ventricular EF of healthy mice is wide-ranging (60–90%) but typically higher than that of the human heart [43–44]. Furthermore, unlike clinical echocardiography, functional indices of heart function in experimental mice may be influenced by anesthesia and usually do not incorporate exercise or pharmacological triggers to evaluate cardiac function under stress. Nonetheless, our echocardiographic findings in this study are statistically robust in demonstrating reversal of adverse functional parameters by hUC-MSC injections and are matched by amelioration of clinically-relevant abnormalities of heart tissue. It should also be noted that evidence of functional benefits of MSC administration have been reported in several clinical trials of ischemic cardiomyopathy [45].

Consistent with these functional abnormalities, our quantitative microscopic analysis of Masson’s trichrome-stain cardiac tissue from the same animals, revealed significant DM-associated increases in interstitial and perivascular fibrosis which were reduced in recipients of hUC-MSCs. Taken together, the functional and structural abnormalities observed in our DM groups are concordant with previous preclinical studies in which significant cardiac dysfunction and myocardial fibrosis were observed in STZ-induced models of DM of 8 weeks [18] and 14 weeks [18] duration. Strikingly, our results indicate that single i.v. injections of hUC-MSCs are capable of potently modulating the underlying pathophysiology of DCM at varying stages of its progression toward severe cardiac dysfunction.

Umbilical cord-derived MSCs are an important member of the growing family of MSC-based therapeutic products and are increasingly gaining attention. Their readily accessible source and their capacity for promotion of tissue repair and immune regulation make them particularly well suited to treating diseases involving tissue degeneration caused by chronic inflammation [46–48]. Based on these properties, hUC-MSCs have been tested in pre-clinical and clinical studies involving a range of chronic disease states including arthritis, stroke, liver disease, diabetes mellitus, systemic lupus erythematosus and cardiovascular disease [49]. In the case of DCM, however, previous preclinical experimental studies of MSCs mainly involved the administration of bone marrow (BM)- and adipose tissue (AT)-derived MSCs [50]. These preclinical experimental studies, as well as one involving the administration of Wharton’s jelly derived-MSCs [51], did not compare the disease progression and effects of MSC administration at both early and later stage of DCM.

Cardiac fibrosis is one of the most common pathological changes in DCM, which eventually leads to cardiac dysfunction and heart failure [52]. In DCM, myocardial fibrosis is a chronic and progressive process characterized by the accumulation of collagen fibers in the myocardium in the setting of prolonged hyperglycemia [53, 54]. Jin et al. demonstrated that adipose-derived MSC administration was beneficial in a rat model of DCM when administered 8 weeks after STZ [55]. Although both interstitial fibrosis was increased by DM at both time-points and was ameliorated by a single, relatively late i.v. injection of hUC-MSCs, it was notable that only peri-vascular fibrosis had worsened significantly between 10 and 18 weeks in DM mice and that this accelerated advancement was effectively prevented by hUC-MSCs.

MicroRNAs are known to be key modulators of the post-transcriptional regulation of genes in all cells types [56]. Current research confirms that miRNAs play important roles in pathological processes in the cardiovascular system, including arrhythmia, cardiac hypertrophy, cardiac fibrosis and myocardial infarction [57]. For this reason, miRNAs have emerged as novel targets for DCM treatment and important tools for disease diagnosis, prevention, and treatment [58]. It is known that MSCs release large amounts of extracellular vesicles (EVs) containing various bioactive molecular including a range of miRNAs and may, therefore, be a potent sources of transferable miRNAs in disease settings [59]. This current study revealed that the expression of miRNA-133a in the hearts of diabetic mice was significantly lower than that in normal mice and that this reduction was attenuated in the groups that received hUC-MSCs at both time points assessed. It has previously been shown that cardiac miR-133a is down-regulated in DM and that miR-133a overexpression in the heart (through cloning the genomic sequence of miR-133a into the alpha-myosin heavy-chain promoter) prevented early cardiac fibrosis in DM mice [60].

The regulation of target genes (mRNAs) by miRNAs occurs at the post-transcriptional level, mainly through the inhibition of their translation to protein. There are many studies confirming that miRNA-133a regulates fibrosis in various organs such as heart, lung, and kidney through downstream mRNAs [61]. Thus, we also examined the intra-cardiac expression of mRNAs encoding the collagen chains COL1A1 and COL3A1 (the former being a known target of miRNA-133a), along with mRNAs encoding other pro-fibrotic proteins, ACTA2(α-SMA), TGFB1(TGF-β), Smad2, Smad3, Smad4, and the anti-fibrotic mediator FGF1. Collagen I and collagen III are the main components of extracellular matrix (ECM), which is central to wound healing and fibrosis [62]. Our results are consistent with the potential role of miR-133a downregulation to enhance the expression of COL1A1(Collagen I) and with the reversal of this pro-fibrotic effect by systemic hUC-MSC therapy. ACTA2(α-SMA) is one of six different actin isoforms involved in smooth muscle contraction. It is considered to be a marker of myofibroblasts, and its gene expression level correlates with the degree of tissue fibrosis [63]. Similarly, the TGF-β/Smad (Smad2, Smad3, Smad4) signaling pathway is an important driver of fibrocyte division and collagen deposition and is an recognized target for regulating fibrosis [64]. In addition to its modulatory effects on fibrosis, FGF1 has also been reported to be a potent insulin sensitizer [65]. Taken together, the results for these mRNA quantification studies were in keeping with a potent anti-fibrotic effect of both earlier and later hUC-MSC injection, resulting in amelioration of the degree of interstitial and perivascular fibrosis and improved cardiac systolic and diastolic function.

While modulation of miRNA expression within the myocardium represents a potential mechanism by which hUC-MSCs may directly mediate anti-fibrotic effects, our observations could also be explained by an indirect mechanism through the well-documented anti-inflammatory properties of MSCs [66]. Indeed, DM is known to be a pro-inflammatory state and multiple inflammatory pathways and mediators have been implicated in the pathophysiology of DCM [67]. In keeping with this, we observed that diabetic mice had progressively increasing circulating levels of the archetypal pro-inflammatory cytokines IL-6, TNF and IL-1β following 10 and 18 weeks of hyperglycemia which were corrected toward non-diabetic levels by hUC-MSC injections, albeit to a lesser extent at 18 weeks. This pattern was mirrored by blood levels of the pro-fibrotic cytokine TGFβ1. In contrast, our tissue-level analysis indicated a relatively subtle inflammatory process with 1.5-3-fold increases in mRNA expression of a limited number of cytokines at each time-point and no evidence of overt infiltration of the myocardium by macrophages or T cells. Nonetheless, the mRNA signatures of cardiac inflammation were prevented or significantly reduced by hUC-MSC administration and, at the later time-point, an increase in the anti-inflammatory cytokine IL-10 was observed. As IL-6 has been shown to promote LV hypertrophy and myocardial fibrosis [68], it is of specific interest that tissue expression of IL-6 mRNA and protein was increased at both time-points in diabetic mice and was suppressed by hUC-MSC injection. Overall, our results do indicate the hUC-MSC injections were associated with systemic and localized anti-inflammatory effects which may have played an important role in ameliorating cardiac fibrosis in diabetic mice. These effects were more potent at the earlier time-point while fibrosis was similarly reduced at both time-points. Importantly, while inflammation is a critical driver of fibrosis in many disease states, targetable pro-fibrotic mechanisms also occur directly within tissue fibroblasts, endothelial cells and other parenchymal cell types [69]. Although further mechanistic experiments are needed, we interpret the results of this study as providing evidence for a complex mechanism of action of hUC-MSCs in experimental DCM that may involve both direct anti-fibrotic effects through modulation of miRNA expression as well as suppression of hyperglycemia-driven systemic and localized inflammatory pathways [70–72].

## Conclusion

The results we present here have demonstrated that single i.v. injections of hUC-MSCs improved cardiac function and myocardial fibrosis in male mice with diabetes of shorter and longer duration. These findings extend the body of evidence to support the clinical use of hUC-MSCs for prevention or treatment of symptomatic DCM. Mechanistically, our findings are consistent with a mode of action whereby systemically administered hUC-MSCs either directly or indirectly reverse diabetes-associated cardiac fibrosis through restoring intra-myocardial expression of the anti-fibrotic miRNA, miR-133a in association with systemic and localized anti-inflammatory effects.

### Electronic supplementary material

Below is the link to the electronic supplementary material.

Supplementary Material 1

## Data Availability

The data that support the findings of this study are available from the corresponding author upon reasonable request.

## Declarations

### Research in context

What is already known about this subject?

Diabetic cardiomyopathy (DCM) is a severe and common complication of prolonged diabetes mellitus and is associated with myocardial fibrosis.

Mesenchymal stromal cells (MSCs) have been reported to improve cardiac fibrosis in experimental models of diabetes.

Potential mechanisms of action of MSCs in diabetic complications include modulation of abnormal miRNA expression and suppression of pro-fibrotic inflammation.

What is the key question?

Does intravenous injection of human umbilical cord-derived mesenchymal stromal cells (hUC-MSCs) reduce cardiac fibrosis in association with modulation of intra-myocardial microRNA (miRNA) expression following shorter and longer duration of diabetes?

What are the new findings?

1, Single intravenous injections of hUC-MSCs administered following 8 or 16 weeks of established diabetes reduced the severity of cardiac functional abnormalities and fibrosis detected 2 weeks later (10 and 18 weeks).

2, At earlier and later time-points, diabetic cardiomyopathy was associated with decreased intra-cardiac expression of the anti-fibrotic miRNA-133a and concomitant increased expression of its target mRNA, COL1A1 and other markers of fibrosis. These abnormalities were potently reversed in diabetic animals that received hUC-MSC injection.

3, Diabetes was also associated with evidence of systemic inflammation as well as increased intra-cardiac expression of some cytokines at both time-points. These abnormalities were more potently reversed by hUC-MSC injection at earlier compared to later time-point.

How might this impact on clinical practice in the foreseeable future?

Provide preclinical evidence for the clinical application of human umbilical cord mesenchymal stromal cells in the treatment of diabetes heart disease in the future.

## Abbreviations

AD-MSCs: Adipose-Derived Mesenchymal Stem Cells
AGS: Animal General Scoring
ANOVA: Ordinary one-way analysis of variance
BM-MSCs: Bone Marrow Mesenchymal Stem Cells
CO: Cardiac output
CVF: Collagen volume fraction
DCM: Diabetic cardiomyopathies
DM: Diabetes mellitus
*E/A*: The ratio of peak early diastolic filling velocity to late atrial filling velocity
EVs: Extracellular vesicles
FBG: Fasting blood glucose
FS: Short axis fractional shortening
H&E: Hematoxylin and eosin
HF: Heart failure
HR: Heart rate
hUC-MSCs: Human umbilical cord mesenchymal stromal cells
ISCT: International Society for Cellular Therapy
IVRT: Isovolumic relaxation time
LVAWd: Left ventricular anterior wall thickness at end-diastole
LVAWs: Left ventricular anterior wall thickness at end-systole
LVEF: Left ventricular ejection fraction
LVIDd: Left ventricular internal diameter at end-diastole
LVIDs: Left ventricular internal diameter at end-systole
LVPWd: Left ventricular posterior wall thickness at end-diastole
LVPWs: Left ventricular posterior wall thickness at end-systole
LVW: Left ventricular weight
miRNA: microRNA
PCR: Polymerase chain reaction
PVCA/LA: The ratio of perivascular collagen area to luminal area
SPF: Specific pathogen free
STZ: Streptozotocin
SV: Stroke volume
WJ-MSCs: Wharton Jelly Cells
