# Supplementary material for: Human umbilical cord-derived mesenchymal stromal cells improve myocardial fibrosis and restore miRNA-133a expression in diabetic cardiomyopathy
Source: Stem Cell Res Ther. 2024 Apr 24;15:120. doi: 10.1186/s13287-024-03715-2 (PMC11040946; doi:10.1186/s13287-024-03715-2)
Supplement: Supplementary file 1 — Supplementary Material 1 [file 13287_2024_3715_MOESM1_ESM.pdf]

Supplemental Table 1: Animal General Distress Scoring Parameters

Parameter	Grading	Scoring criteria	Score
Fur aspect	0	Normal, Actively grooming	
	1	General lack of grooming/dulling of hair coat	
	2	Coat staring, rough coat, ocular and nasal porphyrin staining	
	3	Piloerection	
Natural behavior /activity	0	Normal level of activity	
	1	Minor Changes, less active	
	2	Less mobile and alert, isolated from others or no nesting behavior	
	3	Vocalization, self-mutilation, restless or still even if disturbed	
Posture	0	Normal	
	1	Slightly hunched when moving freely	
	2	Hunched with stiff movement posture/writhing/hoping	
	3	Hunched and belly pressing. Reluctant to move	
Provoked behavior	0	Normal	
	1	Mild depression or exaggerated responses when disturbed	
	2	Moderate changes in expected provoked behavior. Slow reactions when provoked	
	3	Reacts violently or very weak, pre-comatose, reluctant to move even if disturbed	
Body weight (% weight loss from original)	0	% weight loss: 0-5%	
	1	% weight loss: >5-10%	
	2	% weight loss: >10-15%	
	3	% weight loss :>15%	
Hydration status	0	Normal	
	1	Skin pinch slow	
	2	Skin pinch persistent	
Respiratory signs	0	Normal Respiratory Pattern	
	1	Mild tachypnoea	
	2	Moderate tachypnoea	
	3	Marked respiratory distress/tachypnoea /cyanosis/abdominal breathing	

Supplemental Table 2 The miRNA mature sequences and miRNA primer F sequences

miRNA name	miRNA mature sequences	primer F sequences
let-7f	5′-UGAGGUAGUAGAUUGUAUAGUU-3′	5′-GGCGGTGAGGTAGTAGATTGTATAGTT-3′
miR-26a	5′-UUCAAGUAAUCCAGGAUAGGCU-3′	5′-CCGTTCAAGTAATCCAGGATAGGCT-3′
miR-29a	5′-UAGCACCAUCUGAAAUCGGUUA-3′	5′-AACCGGTAGCACCATCTGA-3′
miR-29b	5′-UAGCACCAUUUGAAAUCAGUGUU-3′	5′-TAGCACCATTTGAAATCAGTGTT-3′
miR-29c	5′-UAGCACCAUUUGAAAUCGGUUA-3′	5′-TAGCACCATTTGAAATCGGTTA-3′
miR-34a	5′-UGGCAGUGTCUUAGCUGGUUGU-3′	5′-ACACCATGGCAGTGTCTTAG-3′
miR-133a	5′-UUUGGUCCCCUUCAACCAGCUG-3′	5′-TTTGGTCCCCTTCAACCAGCTG-3′
miR-155	5′-UUAAUGCUAAUUGUGAUAGGGGU-3′	5′-GGCGTTAATGCTAATTGTGATAGGGGT-3′
miR-326	5′-CCUCUGGGCCCUUCCUCCAGU-3′	5′-CTCTGGGCCCTTCCTCCA-3′

Supplemental Table 3 The mRNA primer F and R sequences

mRNA name	primer F sequences	primer R sequences
β-actin	5′-GTCCACCGCAAATGCTTCTA-3′	5′-TGCTGTCACCTTCACCGTTC-3′
ACTA2(α-SMA)	5′-TGCTGGACTCTGGAGATGGTGTG-3′	5′-CGGCAGTAGTCACGAAGGAATAGC-3′
COL1A1(collagen I)	5′-TGTGGGCATCAATGGATTTGG-3′	5′-CAGATCACGTCATCGCACAAC-3′
COL3A1(collagen III)	5′-TTGAAGGAGGATGTTCCCATCT-3′	5′-ACAGACACATATTTGGCATGGTT-3′
Smad2	5′-AAGCCATCACCACTCAGAATTG-3′	5′-CACTGATCTACCGTATTTGCTGT-3′
Smad3	5′-TCCTGGCTACCTGAGTGAAGA-3′	5′-GTTGGGAGACTGGACGAAAA-3′
Smad4	5′-AGGACAGAAGCCATTGAG-3′	5′-CGATGACACTGACGCAAA-3′
TGFB1(TGF-β)	5′-CCAAGGAGACGGAATACAGG-3′	5′-GTGTTGGTTGTAGAGGGCAAG-3′
FGF1	5′-CTCCCGAAGGATTAAACGACG-3′	5′-GTCAGTGCTGCCTGAATGCT-3′

Supplemental protocols

H&E staining protocol:

H&E staining was performed on sections according to the standard protocol of the H&E staining kit(Service biological science and technology Ltd.,Wuhan,China).Sections were fixed in 4% paraformaldehyde, washed with ddH2O, stained with hematoxylin, differentiated in 1% HCl ethanol, washed thoroughly, soaked in ammonia until nuclei became blue in color, and stained in eosin. Then, the sections were dehydrated with graded alcohols, cleared twice in xylene, and then sealed with neutral resin. Stained sections were photographed with a dp74 light microscope (Olympus) for observation and pictures were taken randomly in a blinded fashion.

Masson's staining protocol:

Masson's trichrome staining was performed on sections according to the standard protocol of the Masson staining kit(Service biological science and technology Ltd.,Wuhan,China).After the sections were deparaffinized to water, hematoxylin stained nuclei Ponceau red stained, phosphomolybdic acid treated, aniline blue stained, differentiated, neutral gum mounted slides after dehydration. Stained sections were photographed with a dp74 light microscope (Olympus) for observation and pictures were taken randomly in a blinded fashion.

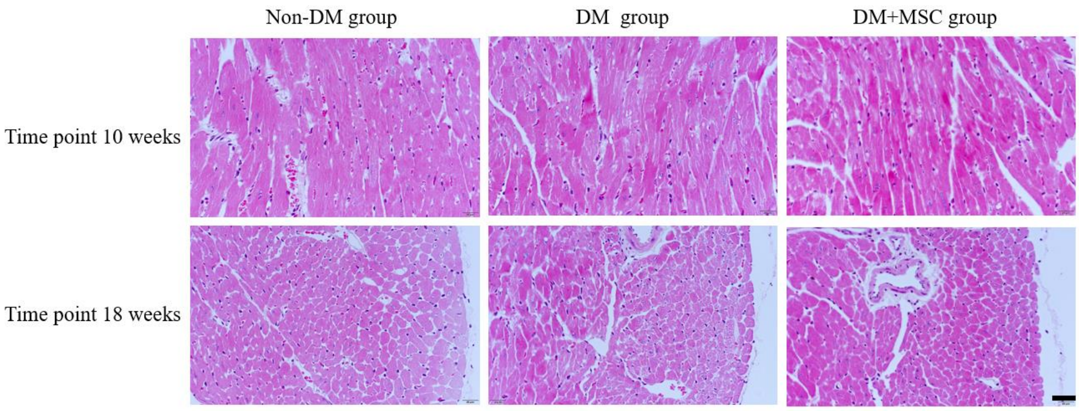

Supplemental Figure 1A: Representative examples of photomicrographs of H&E-stained sections of cardiac tissue from three groups of experimental mice from experiments of 10 weeks and 18 weeks duration. Scale bar = 20 μm, Magnification =200×.

Abbreviations: Non-DM = no diabetes; DM = diabetes mellitus with saline injections; DM+MSC = diabetes mellitus with hUC-MSC injections.

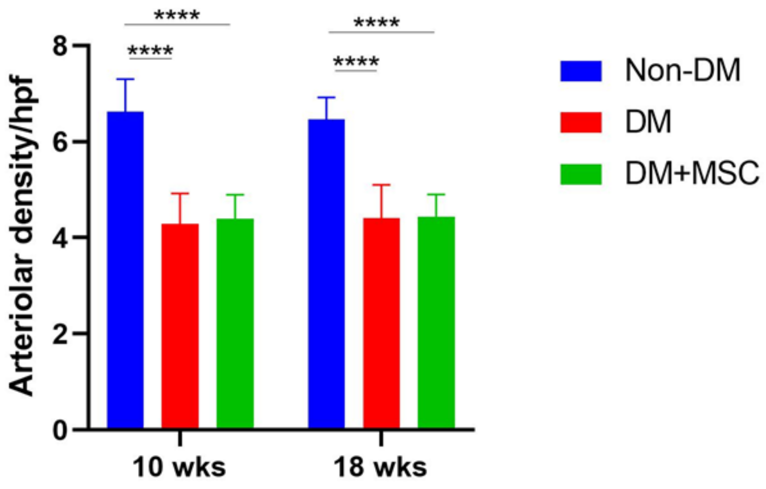

Supplemental Figure 1B: Arteriole density per high power field (hpf) in H&E-stained. n=6 each group, with each mouse being included in 3 sections. From each section, 6 fields were randomly selected for statistical analysis (total 18 fields per heart). Statistical analysis was performed by one-way ANOVA.

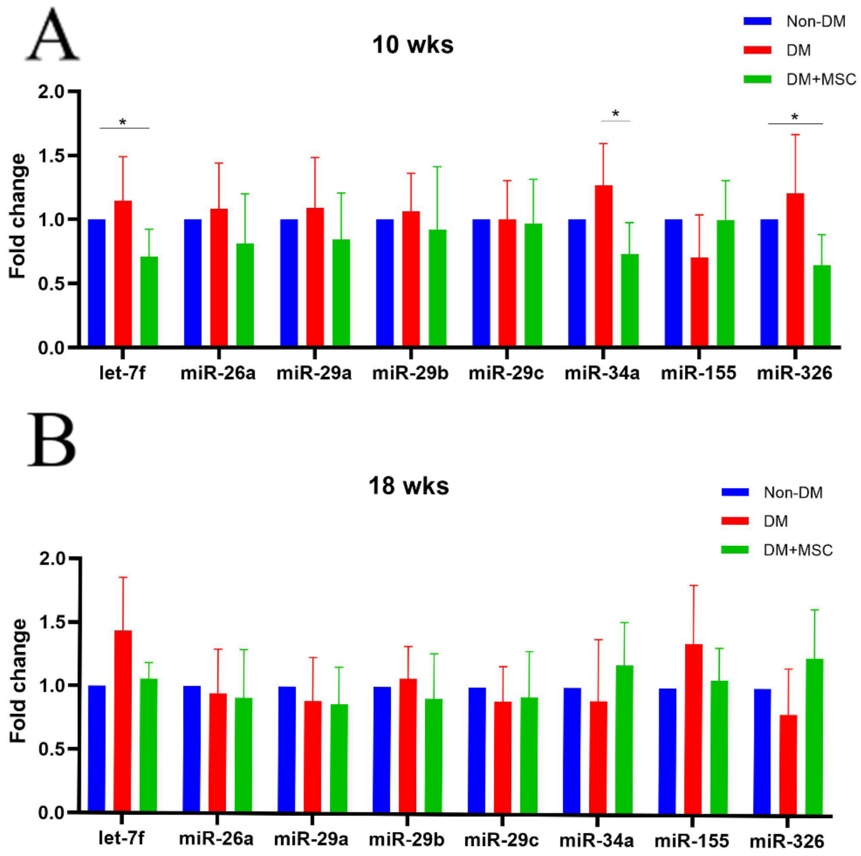

Supplemental Figure 2: A-B. Graphs of quantitative RT-PCR analyses of the relative expression of 8 selected miRNAs in heart tissue of three groups of mice each from experiments of 10 weeks (**A**) and 18 weeks (**B**) duration. Levels for DM and DM+MSC groups are expressed as fold change relative to the Non-DM group. All values are expressed as mean ± SD of n=6 biological replicates. Statistical analysis was performed by one-way ANOVA. *P<0.05. Abbreviations: Non-DM = non-diabetic group; DM = diabetes mellitus with injection of saline; DM+MSC = diabetes mellitus with injection of hUC-MSCs.

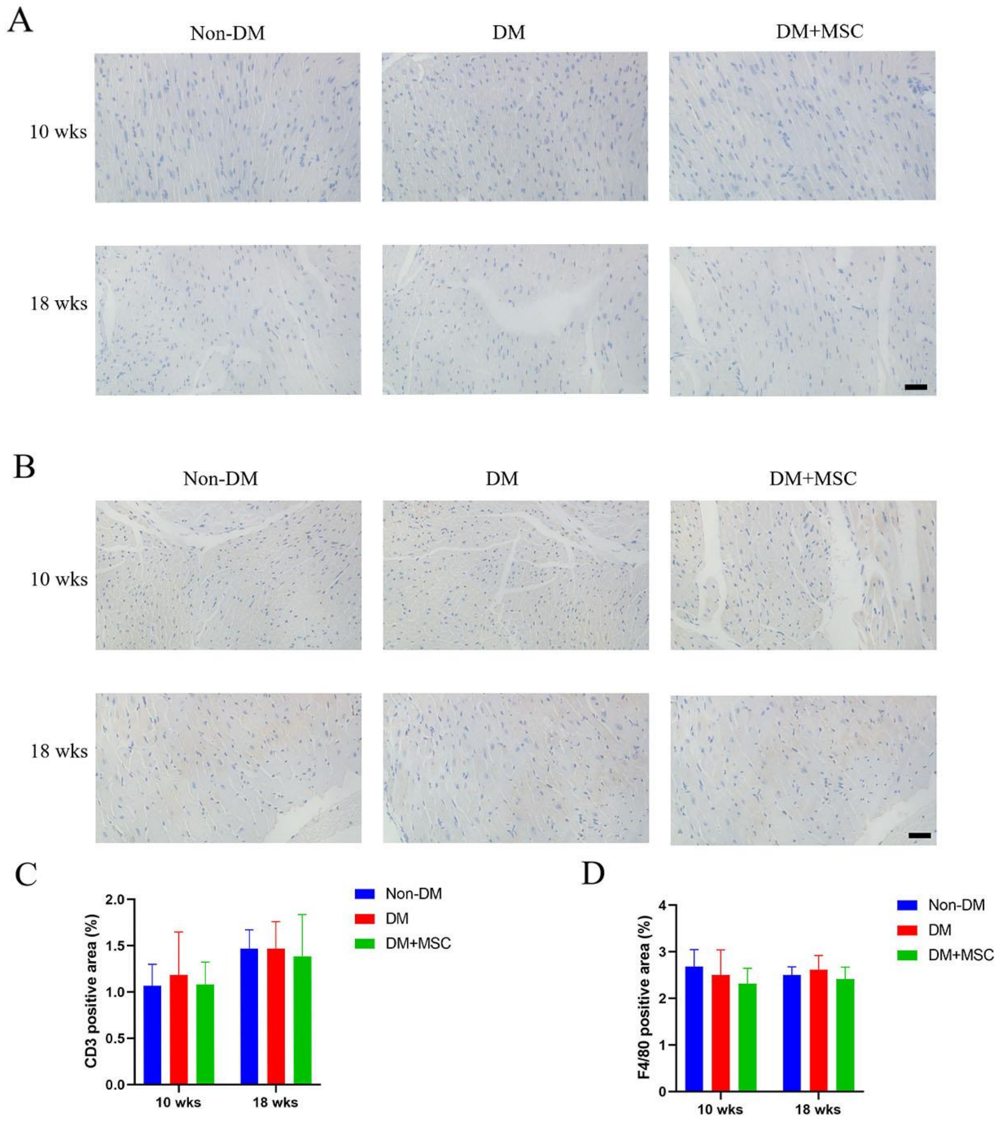

Supplemental Figure 3: Immunohistochemical experimental results for CD3 (**A**) and F4/80 (**B**). For quantification of antibody positive area for CD3 (**C**) and F4/80 (**D**) were calculated for each field as the ratio of the brown-stained area to the total area. The experimental groups consisted of 6 mice each, with each mouse being included in 3 sections. From each section, 6 fields were randomly selected for statistical analysis (total 18 fields per heart). The final positive expression area values, expressed as %, were derived for each heart from the average values of all analyzed fields. Statistical analysis was performed by one-way ANOVA.
